# The utility of explainable AI for MRI analysis: Relating model predictions to neuroimaging features of the aging brain

**DOI:** 10.1162/imag_a_00497

**Published:** 2025-02-27

**Authors:** Simon M. Hofmann, Ole Goltermann, Nico Scherf, Klaus-Robert Müller, Markus Löffler, Arno Villringer, Michael Gaebler, A. Veronica Witte, Frauke Beyer

**Affiliations:** Department of Neurology, Max Planck Institute for Human Cognitive and Brain Sciences, Leipzig, Germany; Neural Data Science and Statistical Computing, Max Planck Institute for Human Cognitive and Brain Sciences, Leipzig, Germany; Department of Electrical Engineering and Computer Science, Technical University Berlin, Berlin, Germany; Max Planck School of Cognition, Leipzig, Germany; Institute of Systems Neuroscience, University Medical Center Hamburg-Eppendorf, Hamburg, Germany; Center for Scalable Data Analytics and Artificial Intelligence (ScaDS.AI) Dresden/Leipzig, Germany; BIFOLD - Berlin Institute for the Foundations of Learning and Data, Berlin, Germany; Department of Artificial Intelligence, Korea University, Seoul, Korea (the Republic of); Max Planck Institute for Informatics, Saarbrücken, Germany; Institute for Medical Informatics, Statistics and Epidemiology; University of Leipzig, Leipzig, Germany; Leipzig Research Centre for Civilization Diseases (LIFE), Leipzig, Leipzig, Germany; Clinic for Cognitive Neurology, University Hospital Leipzig, Leipzig, Germany; Center for Stroke Research, Charité – Universitätsmedizin Berlin, Berlin, Germany; Berlin School of Mind and Brain, Humboldt-Universität zu Berlin, Berlin, Germany; Bordeaux Population Health Center, University of Bordeaux, Bordeaux, France

**Keywords:** deep learning, brain age, explainable AI, cortical thickness, subcortical volume, cerebral small vessel disease

## Abstract

Deep learning models highly accurately predict brain age from MRI but their explanatory capacity is limited. Explainable artificial intelligence (XAI) methods can identify relevant voxels contributing to model estimates, yet they do not reveal which biological features these voxels represent. In this study, we closed this gap by relating voxel-based contributions to brain-age estimates, extracted with XAI, to human-interpretable structural features of the aging brain. To this end, we associated participant-level XAI-based relevance maps extracted from two ensembles of 3D-convolutional neural networks (3D-CNNs) that were trained on T1-weighted and fluid-attenuated inversion recovery images of 1855 participants (age range 18–82 years), with regional cortical and subcortical gray matter volume and thickness, perivascular spaces (PVS), and water diffusion-based fractional anisotropy of major white matter tracts. We found that all neuroimaging markers of brain aging, except for PVS, were highly correlated with the XAI-based relevance maps. Overall, the strongest correlation was found between ventricular volume and relevance (*r*= 0.69), and by feature, temporal-parietal cortical thickness and volume, cerebellar gray matter volume, and frontal-occipital white matter tracts showed the strongest correlations with XAI-based relevance. Our ensembles of 3D-CNNs took into account a plethora of known aging processes in the brain to perform age prediction. Some age-associated features like PVS were not consistently considered by the models, and the cerebellum was more important than expected. Taken together, we highlight the ability of end-to-end deep learning models combined with XAI to reveal biologically relevant, multi-feature relationships in the brain.

## 1 Introduction

Deep neural networks (DNNs) have become a valuable asset for the analysis of neuroimaging data, facilitating the classification of brain-related diseases (e.g., Alzheimer or multiple sclerosis;Vieira et al., 2017;Zhang et al., 2018) and the estimation of continuous variables such as brain age (BA;Ball et al., 2021;Bashyam et al., 2020;Cole et al., 2017;Dinsdale et al., 2021;Feng et al., 2020;Peng et al., 2021) from minimally processed magnetic resonance images (MRIs), avoiding a priori feature selections. While DNNs have shown to be highly accurate prediction models in many domains, their decision process is difficult to interpret due to their high complexity (also known as the black box problem). Only recently, the imaging community started to employ algorithms that partially explain the internal decision process of these models (Böhle et al., 2019;Eitel et al., 2019;Hofmann et al., 2022;Levakov et al., 2020;Thomas et al., 2019).

### 1.1 Challenges in interpreting relevance maps

The majority of these explainable artificial intelligence (XAI) algorithms generate explanations*post hoc*(Ras et al., 2022) in the form of relevance maps (also heatmaps, or saliency maps), highlighting pixels or voxels which have been relevant for the prediction of the model. These relevance-based explanations became popular since they appeal to human intuition. Common benchmarks for both prediction models and XAI algorithms often contain natural images of, for example, cats, cars, and other familiar objects. When a prediction model correctly classifies samples from such familiar categories, the corresponding relevance maps highlight particular object-related features that usually appeal to our understanding of the corresponding class (e.g., the eyes and ears of a cat). However, in contrast to the application of XAI on natural images, our intuition regarding the relevance maps of MRI-based predictions is limited, even for neuroimaging experts. Moreover, there is a danger of human bias when selectively interpreting relevance maps (confirmation bias;Adebayo et al., 2020;Bacon, 1620;Ghassemi et al., 2021;Lakens, 2022). Therefore, there is a need for analyzing relevance maps quantitatively and relating them to human interpretable features.

### 1.2 Linking relevance maps to imaging markers

One way to achieve this is to aggregate relevance over regions derived from brain atlases. For instance,Eitel et al. (2019)used gray and white matter (WM) atlases to determine which brain regions were the most relevant for their deep learning model to accurately diagnose multiple sclerosis. Another way is to overlap explanation maps with spatial maps of other neuroimaging markers, which also capture distributed neural-structural properties. This way, in our previous work (Hofmann et al., 2022), we could show that white matter hyperintensities (WMHs), a common marker of cerebral small vessel disease (cSVD;Cuadrado-Godia et al., 2018;Li et al., 2018) in WM, were relevant for our FLAIR-based BA prediction model as indicated by the XAI method Layer-wise Relevance Propagation (LRP;Lapuschkin et al., 2019). LRP takes a prediction and propagates it back through the DNN layer-by-layer, identifying the relevant contributions to the prediction up until the input (here the MRI). WMH only partially explained the highly accurate BA predictions of our model. That is, only a fraction of the total relevance scores were captured by voxels containing WMHs. Additionally, on visual inspection, we found that higher relevance reflected gray matter atrophy around the ventricles but did not quantify this yet. In summary, while previous studies demonstrated that XAI methods can provide initial information about the decision process of deep-learning-based BA models (Hofmann et al., 2022;Levakov et al., 2020), we so far only partially understand which neural features the highlighted areas represent.

### 1.3 Study objectives and hypotheses

In this study, we investigated the contribution of well-known quantifiable imaging markers of age-related biological changes to highly precise deep-learning-based BA estimates. To this end, we tested gray matter (GM) properties, including volume (GMV) in the cortex, in subcortical regions, the cerebellum and brainstem, cortical thickness (CT), and ventricular volume as imaging markers of GM atrophy, a prominent feature of brain aging (Fjell & Walhovd, 2010). Moreover, we examined the WM microstructure of neural fiber bundles represented in fractional anisotropy measures (FA;Basser & Pierpaoli, 2011), which is known to reflect age-related changes in the WM organization (Schilling et al., 2022). Finally, we investigated another imaging marker of cSVD, namely perivascular spaces (PVS), which have a characteristic appearance of small, liquid-filled structures parallel to perforating blood vessels (Swieten et al., 1991;Wardlaw et al., 2020). The automatic quantification of PVS is relatively novel, but it has been shown that age is a strong predictor for the presence of PVS (Francis et al., 2019). Based on these associations, we hypothesized that higher relevance scores (i.e., higher predicted BA) are associated with smaller CT in cortical regions, and lower GMV of both cortical and subcortical regions, as well as of cerebellum and brainstem. Moreover, we expected higher relevance values to overlap with PVS in deep WM around the basal ganglia and in the whole brain and to correlate with lower FA values in WM tracks. We emphasize that while classical approaches (e.g., region-, voxel-, or vertex-wise analysis) have been effective in understanding aging processes in the brain, the primary goal of this study is not to compare DNNs directly with these methods. Instead, our aim is to apply XAI techniques, here LRP, to help understand how deep learning models utilize neural features to predict brain age. This approach is motivated by the desire to bridge the gap between high predictive accuracy of DNNs and their current lack in interpretability.

## 2 Methods

### 2.1 Participants

Participants took part in the baseline assessment of the population-based cohort LIFE-Adult study (Loeffler et al., 2015), in which they underwent extensive clinical screenings, including measures of height, weight, blood pressure, blood-based biomarkers, neuroimaging, cognitive performance, and surveys on mental health and lifestyle (for further information, refer toLoeffler et al., 2015). Among the more than 10,000 subjects enrolled in the LIFE Adult study, 2637 participants underwent a baseline 1-hour brain MRI recording session. Out of those individuals who received MR-scans, 621 participants were excluded primarily due to pathologies, resulting in 2016 subjects for further analysis (age range of 18–82 years, mean age = 57.32, median age = 63.0; n_females_= 946). Among the 621 participants excluded, criteria partially overlapped: Exclusion criteria included previous strokes (n = 54), excessive brain lesions evaluated by trained medical professionals (n = 114), including white matter hyperintensities (WMH) with a Fazekas score of 3 (n = 44), radiological diagnosis of brain tumors (n = 22), diagnosis of multiple sclerosis (n = 5), epilepsy (n = 27), recent cancer treatment (n = 109), centrally active medication (n = 275), cognitive impairments indicated by an MMSE score < 26 (n = 80), and inadequate quality MRIs (failing a visual quality check, e.g., motion artifacts, n = 41).

### 2.2 Ethics statement

The LIFE-Adult study received approval from the University of Leipzig’s ethics committee and was carried out in accordance with the Declaration of Helsinki. All participants provided written informed consent.

### 2.3 MRI data acquisition

MRI data were obtained on a 3T Siemens Verio scanner, equipped with a 32-channel head coil. In this study, we made use of four MRI-sequences commonly utilized in clinical settings: i) Structural T1-weighted images were captured using a MPRAGE-sequence (T1w; 1 mm isotropic voxels, 176 slices, TR = 2300 ms, TE = 2.98 ms, TI = 900 ms, field of view 256 × 240 × 176 mm^3^). ii) Fluid-attenuated inversion recovery images (FLAIR; 1 × 0.49 × 0.49 mm^3^voxels, sagittal orientation, 192 slices, TR = 5000 ms, TE = 395 ms, TI = 1800 ms, field of view 250 × 250 × 192 mm^3^). iii) Diffusion-weighted images (DWI; double spin-echo sequence, voxel size = 1.7 × 1.7 × 1.7 mm^3^, TR = 13.8 ms, TE = 100 ms, field of view 220 × 220 × 123 mm^3^, matrix = 128 × 128, maximum*b*-value = 1000 s/mm^2^in 60 directions, and 7 volumes with*b*-value = 0 s/mm^2^). iv) Susceptibility-weighted images (SWI; T2*-weighted pulse sequence, voxel size = 0.8 × 0.7 × 2.0 mm^3^, 64 slices, TR = 28 ms, TE = 20 ms, field of view 230 × 173 × 128 mm^3^, sagittal orientation).

### 2.4 MRI preprocessing

#### 2.4.1 Cortical and subcortical gray matter metrics

T1w images were processed with FreeSurfer v.5.3.0. We used cortical surface parcellations for relevance extraction and to determine metrics for both cortical thickness (CT) and cortical gray matter volume (GMV) according to the Desikan-Killiany-Tourville atlas (DKT;Klein & Tourville, 2012). Similarly, FreeSurfer’s automated subcortical segmentation procedure (Fischl et al., 2002) was employed to obtain subcortical GMV bilaterally (putamen, nucleus accumbens, hippocampus, amygdala, globus pallidus, caudate, thalamus) and 4 cerebrospinal fluid (CSF) volumes (lateral ventricle, inferior lateral ventricle, third ventricle, and CSF), brainstem volume, and WM and GM volume of the cerebellum. Freesurfer results were visually checked according toKlapwijk et al. (2019)which led to the exclusion of 24 individuals from cortical and subcortical analyses mostly based on segmentation errors due to high head motion.

#### 2.4.2 Identification of perivascular spaces

To detect perivascular spaces (PVS), the SHIVA-PVS toolbox was used (Boutinaud et al., 2021;https://github.com/pboutinaud/SHIVA_PVS; accessed in March 2024). This toolbox utilizes a deep-learning-based algorithm designed for the detection and segmentation of PVS. It is constructed upon a 3D-convolutional neural network (CNN)-based autoencoder, including a U-shaped network (U-Net;Ronneberger et al., 2015) trained on either T1w or both T1w and FLAIR co-registered images. We employed the multimodal T1-FLAIR model to segment PVS for each subject in the individual’s FreeSurfer space. The PVS segmentation failed in four individuals who were excluded for further analysis. The resulting PVS probability maps were thresholded at 0.5. In order to make sure we captured the relevance attributed to the border region of the PVS, we additionally created maps in which we dilated PVS by one and two voxels, respectively. We used whole-brain PVS segmentations as well as PVS masked around the basal ganglia (defined using the ATAG atlas, dilated by 5 voxels,https://www.nitrc.org/projects/atag), because of their larger size and more reliable detection (Boutinaud et al., 2021).

#### 2.4.3 Fractional anisotropy

DWI were processed using a customized pipeline, including the in-house software LIPSIA, MRTRX v.3.0 and the FMRIB Software Library FSL v.5.0.9 (Jenkinson et al., 2012;Lohmann et al., 2001;Tournier et al., 2019). Initially, the raw data for each participant underwent denoising and the reference (B0) images were processed with Gibbs unringing to improve data quality. Skull-stripping was performed using the FSL function BET, and outliers were replaced using FSL eddy outlier replacement. Finally, motion correction and distortion correction were performed and the B0 image was coregistered to the T1w images within LIPSIA. After upsampling the data to 1 mm, diffusion tensor fitting was performed in LIPSIA. The resulting FA image was coregistered to the MNI space using ANTs v.0.4.2 (Avants et al., 2008) in Python v.3.10 and thresholded at ≥0.2 to exclude extreme cross-subject variability within small individual WM tracts and to mitigate misalignments during registration. We then parcellated the FA maps into 48 regions using the DTI-based WM atlas JHU (Mori et al., 2005) and calculated the mean FA for each region. FA images were not available in 17 individuals.

### 2.5 Explainable deep learning predictions of brain age

#### 2.5.1 Prediction models

Brain age (BA) prediction models were based on a multi-level ensemble (MLENS) architecture consisting of three sub-ensembles, each built on a specific MRI sequence (T1w, FLAIR, SWI). Each sub-ensemble comprised ten independently trained base models, each of which was constructed using a 3D-CNN implemented in Keras v.2.3.1 (Chollet, 2015). The base model architecture involved five convolutional blocks, followed by leaky rectified linear units (ReLUs) and max-pooling layers. The convolutional blocks were followed by dropout regularization and two fully connected layers, with the output layer bias fixed to the mean age in the dataset. The network was trained to minimize mean squared error (MSE) using the ADAM optimizer. The dataset was divided into training, validation, and test sets in an 8:1:1 ratio, with model performances evaluated on the test set and reported as mean absolute error (MAE) for better interpretability. Subsequently, a linear head model with weight regularization (*L^2^-norm*) was trained on the predictions of the 10 base models per sub-ensemble on the validation set, followed by evaluation on the test set. The resulting predictions from these three sub-ensembles on the test set were then utilized to train an additional head model atop the MLENS using a five-fold cross-validation procedure, thereby obtaining aggregated predictions across all MRI sequences. The mean absolute error (MAE) of the overall MLENS, which was used in this study, was at 3.88 years. The T1 and FLAIR sub-ensemble, which were used to compute relevance maps (see below), were performed with MAE_T1_= 4.31 and MAE_FLAIR_= 4.13 years, respectively. A more detailed description of the model architecture and model performances can be found inHofmann et al. (2022).

#### 2.5.2 Explanation algorithm and relevance maps

We applied layer-wise relevance propagation (LRP;Bach et al., 2015;Lapuschkin et al., 2019;Montavon et al., 2018;Samek et al., 2021) to study which features drive the performance of our BA prediction models. LRP is an algorithm that generates explanatory heat maps in the input space (i.e., relevance maps) of machine-learning models, including non-linear deep learning models. This is achieved by decomposing the prediction*f(x)*of the model*f*with respect to the input*x*into relevance scores*R*. In deep learning models, this is computed layer by layer down to the input space, while satisfying the conservation criterion in each network layer:ΣR=f(x)(for details, seeMontavon et al., 2018). Note, as a consequence of this property, the same prediction*f(x)*(here, the age estimate) for brains and their regions of different sizes leads to different expected relevance scores per voxel. That is, the larger the brain (region), the more the total relevance (ΣR) is spread over voxels. Therefore, for statistical comparisons across participants, we used the sum-relevance over the expected (i.e., mean) relevance to account for brain size. Unlike alternative explanation methods, LRP stands out for its computational efficiency and its ability to capture both local and global feature interactions, which are driving model predictions. In our study, we applied LRP (using the iNNvestigate v.1.08 toolbox, with the “LRPSequentialPresetA” configuration;Alber et al., 2019) on T1 and FLAIR sub-ensembles with ReLU activation functions (for comparability withHofmann et al., 2022), obtaining relevance maps for all subjects and image modalities in the FreeSurfer subject space (native images resampled to a voxel size of 1 × 1 × 1 mm^3^, 256 slices, with a 256 × 256 matrix size and coronal slice orientation). Note that LRP maps were computed for each sub-ensemble separately. Among the 2016 subjects, in 137 and 140 LRP was not performed for the T1 and FLAIR sub-ensembles, respectively, due to data-split reasons; That is, these subject data have not been part of the independent test sets, on which relevance maps were computed. These groups did not differ significantly in age (two-sample t-test: T_T1_= -0.44, p_T1_= 0.6; T_FLAIR_= -0.9, p_FLAIR_= 0.5) and sex (Chi-square_T1_= 0.1, p_T1_= 0.7; Chi-square_FLAIR_= 0.05, p_FLAIR_= 0.8) from the rest of the sample. Relevance maps were also transformed to 2 mm MNI space using a nonlinear registration method based on symmetric normalization implemented in ANTs. As a function of LRP, each voxel is assigned with a relevance score that reflects the voxel’s contribution to the prediction of the model. Since we set the output bias of our networks to the mean age of our sample, negative relevance values represent model evidence in the input toward a younger age (below the mean age in the cohort), and positive values represent evidence toward a higher age (above the mean age in the cohort).

### 2.6 Associations of brain-age relevance maps with imaging features

To evaluate the biological relevance of our deep-learning-based BA model, we examined different aging-related imaging markers derived from T1w, FLAIR, and DWI images (for an overview, seeFig. 1).

**Fig. 1.**
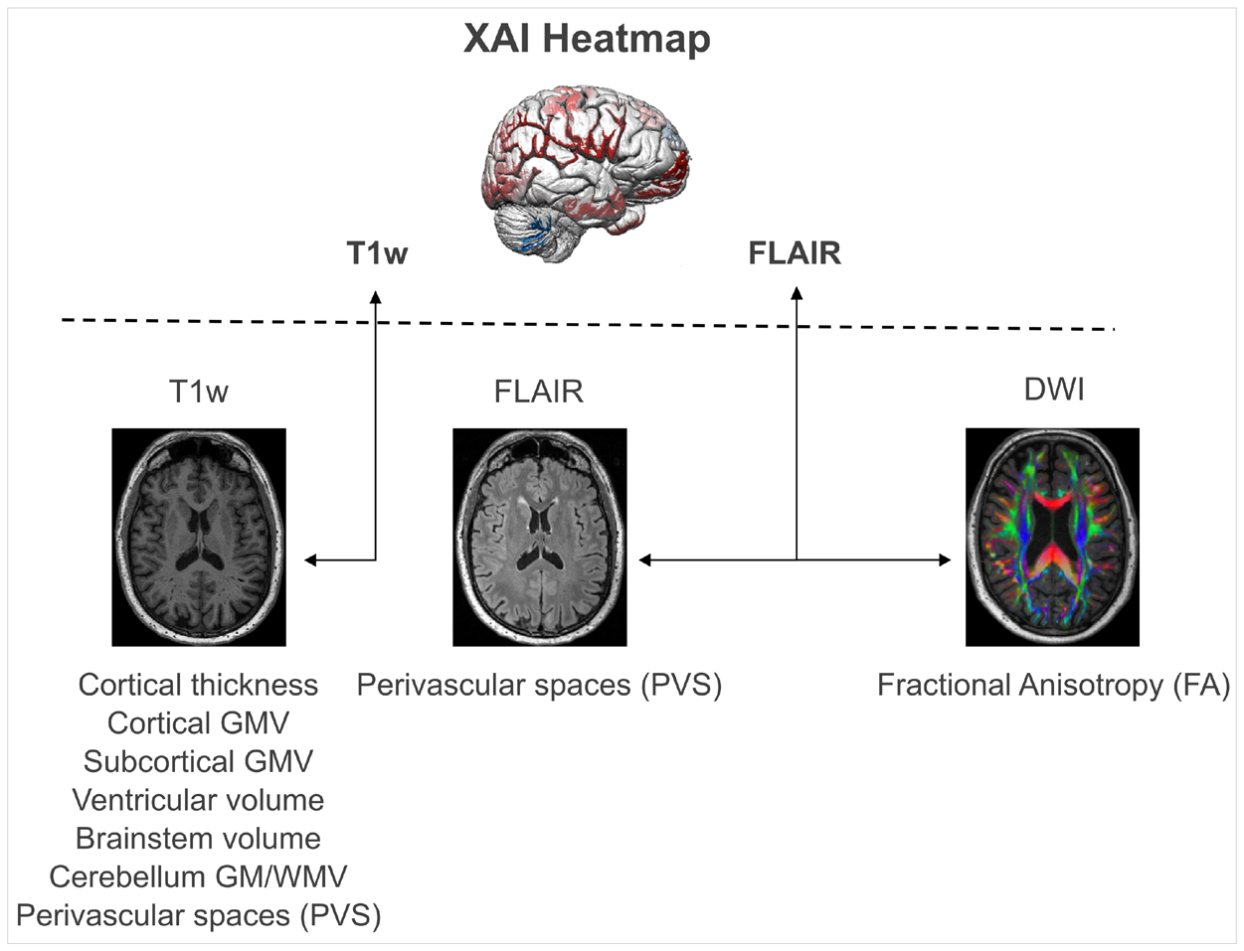
Relationship of aging-related imaging markers and deep-learning-based relevance maps of brain age. A conceptual summary of the goal of the study, in which XAI-extracted features (i.e., relevance maps) underlying deep-learning-based predictions of brain age (top) are associated with common aging-related imaging markers, such as GMV with (bottom). Relevance maps were computed on deep learning models trained on T1w and FLAIR images, respectively. Then, eight different imaging markers were computed on three imaging modalities (T1w, FLAIR, DWI). T1w-based imaging markers were related to T1w-based relevance maps; FLAIR and DWI imaging markers were related to FLAIR-based relevance maps. The contribution of white matter hyperintensities was already tested and reported inHofmann et al. (2022).*XAI: explainable artificial intelligence; GMV: gray matter volume*.

#### 2.6.1 Cortical thickness and volume

For 1855 participants, the regional mean cortical thickness (CT) and volume estimates were correlated with the sum of relevance scores, obtained from the T1w-based LRP relevance maps, in 68 regions (see above) using Pearson’s correlation (coefficient*r*). Bonferroni-correction was used to correct for multiple testing. The sum of relevance, rather than the mean, was used to account for differences in brain/region size (seeSection 2.5.2above). By using the sum of relevance, we ensure that the scores are not influenced by variations in brain or brain region size.

#### 2.6.2 Subcortical, brainstem, cerebellar, and ventricular volumes

For 1855 participants, we correlated subcortical, brainstem, cerebellar, and ventricular volumes in each of their regions defined by various atlases (see above) with the corresponding sum of relevance scores, obtained from T1w-based LRP heatmaps using Pearson’s correlation. We used Bonferroni-correction to adjust for multiple significance testing.

#### 2.6.3 Perivascular spaces

For 1872 participants, we compared the average relevance of PVS voxels in the whole-brain and in areas in and around the basal ganglia (BG) with the average relevance within the brain using one-tailed paired t-tests. Given that the data were positively skewed, we also ran Wilcoxon signed-rank tests to verify the robustness of our results. We used one-tailed tests, because we hypothesized that the LRP value in PVS voxels would be greater than zero, contributing to higher brain-age estimates (Wardlaw et al., 2020). In contrast to the analyses above, here, we used the average relevance, since we were interested in whether the expected relevance was higher in PVS voxels than in the rest of the brain within the same participant. We conducted these tests for relevance values based on T1 and FLAIR sub-ensembles. For T1-relevance scores, we run the tests additionally with dilated PVS maps (dilation*d*in {1, 2} voxels), since PVS are fluid-filled and expected to have zero intensity in T1w imaging, which, in turn, would result in zero relevance scores (i.e., image values of zero do not contribute to a model’s prediction). Thus, to capture potential contributions of PVS to the age predictions, the dilated masks ensure that we include relevance scores at the borders of the PVS into the analysis. We also tested a more conservative approach by comparing the relevance attributed to PVS to any other voxel which is predictive of higher-than-average brain age (i.e., with positive relevance), as used for WMH inHofmann et al. (2022). To verify the relationship between PVS volumes and age, we additionally ran a Pearson correlation between the total number of PVS voxels in participants’ MRIs and their corresponding age at data acquisition.

#### 2.6.4 Fractional anisotropy

To test the relationship between FA values and LRP relevance scores, we performed region-based and voxel-wise analyses in 1872 participants. For the region-based analysis, we correlated the sum of the relevance scores from the FLAIR- and T1-based LRP heatmap with the average FA value for each JHU atlas region. Results were Bonferroni-corrected for multiple comparisons. For the voxel-wise analysis, we correlated the FA values with relevance scores of FLAIR-based LRP heatmaps for each voxel and across subjects in the MNI space. These correlations were motivated by the known sensitivity of FA to microstructural properties of white matter, such as myelination (influencing T1 relaxation time) and tissue integrity (reflected in FLAIR).

## 3 Results

### 3.1 Cortical brain measures

**Fig. 2.**
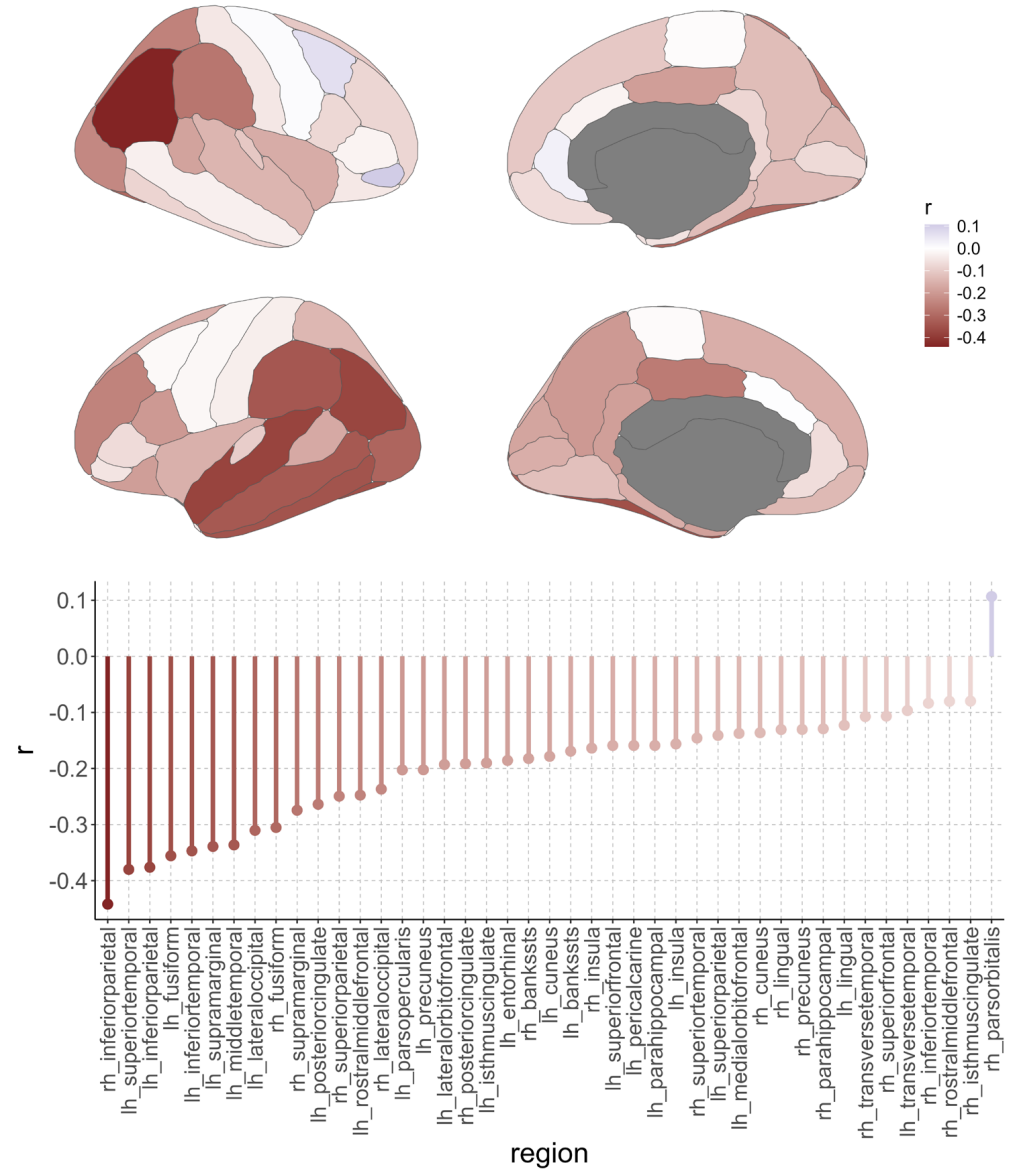
Relationship of cortical thickness with relevance scores from deep-learning-based brain-age predictions across the full lifespan in the LIFE-Adult study (n = 1855).*Top panel*: Region-wise correlation results for the association between cortical thickness and relevance scores, color represents Pearson’s correlation coefficient.*Bottom panel*: Region-wise results for Bonferroni-corrected significant associations between cortical thickness and relevance scores (43 out of 68 regions defined by the DKT atlas).

Out of 68 DKT brain regions (34 per hemisphere), 42 showed a statistically significant negative correlation between the relevance scores and CT, that is, relevance for higher BA was associated with lower CT (mean*r*= -0.20,*r*-range = [-0.44, -0.09]). The strongest negative correlation was present in the inferior-parietal area in the right hemisphere (*r*= -0.44, p < 8.1e-89). Only one area, the right pars orbitalis, showed a positive relationship, that is, relevance for higher brain age was associated with higher CT (*r*= 0.11, p < 0.00024;Fig. 2).

**Fig. 3.**
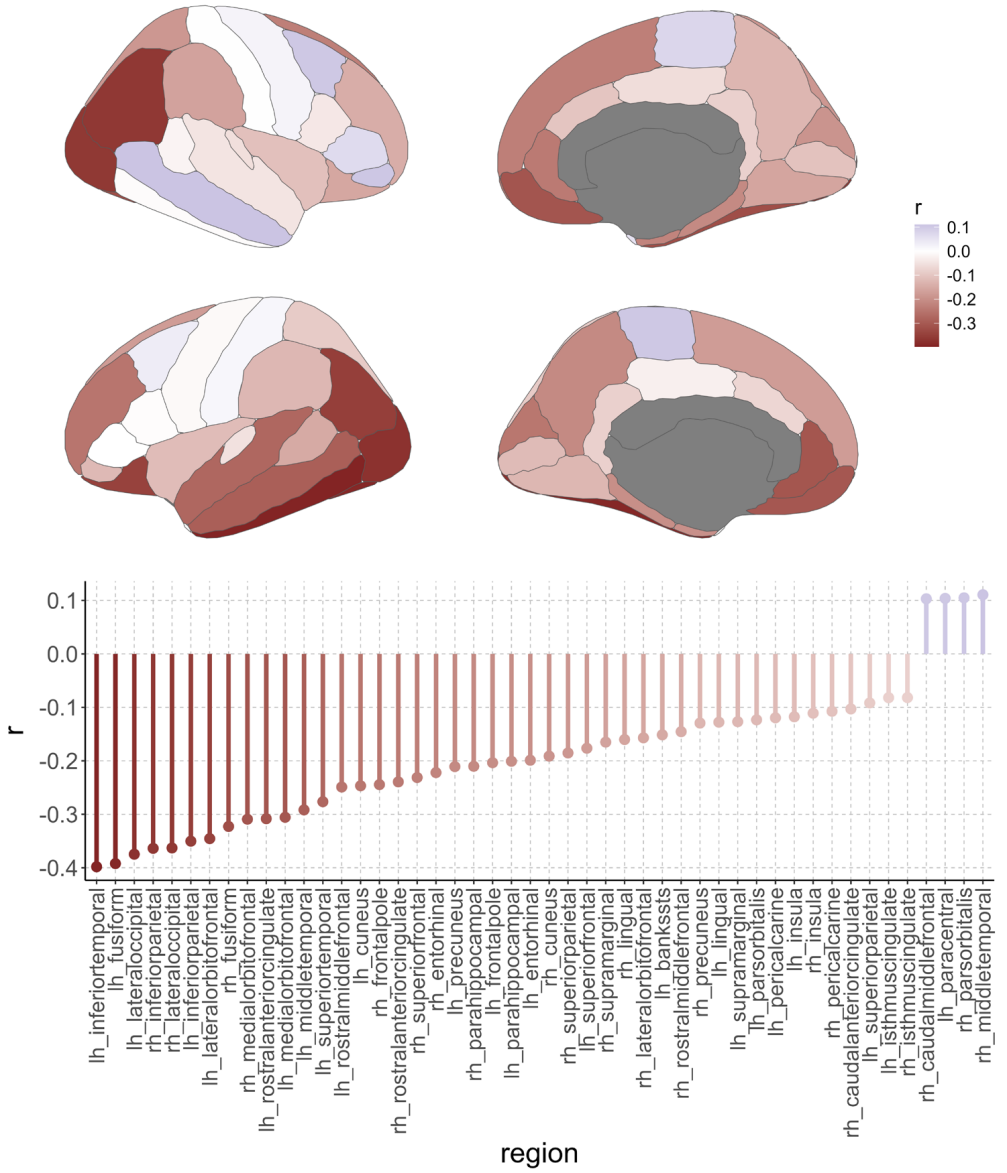
Relationship of cortical gray matter volume with relevance scores from deep-learning-based brain-age predictions across the full lifespan in the LIFE-Adult study (n = 1855).*Top panel*: Region-wise correlation results for the association between cortical gray volume and relevance scores; color represents Pearson’s correlation coefficient.*Bottom panel*: Region-wise results for Bonferroni-corrected significant associations between cortical gray matter volume and relevance scores (48 out of 68 regions defined by the DKT atlas).

Out of 68 DKT brain regions, 44 showed a statistically significant negative relationship between the LRP-based relevance scores and GMV (Bonferroni-corrected, mean*r*= -0. 22,*r*-range = [-0.40, -0.08]). The strongest negative correlation was present in the inferior temporal gyrus in the left hemisphere (*r*= -0.40, p < 1.5e-70). Four areas showed a statistically significant positive relationship (Bonferroni-corrected, mean*r*= 0.11,*r*-range = [0.10, 0.11]). The strongest positive correlation was present in the middle temporal gyrus in the right hemisphere (*r*= 0.11, p < 0.0001;Fig. 3).

### 3.2 Subcortical brain measures

**Fig. 4.**
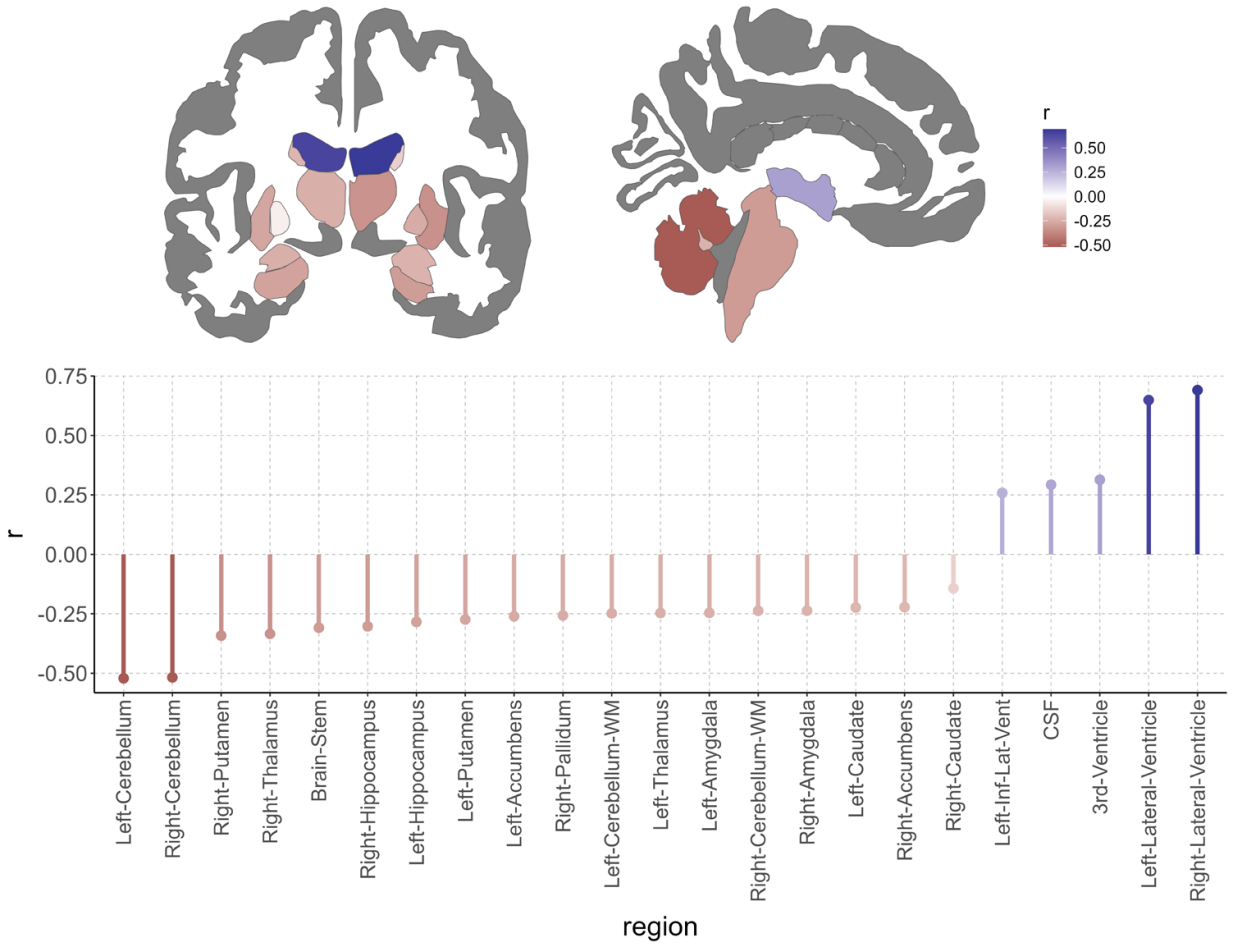
Relationship of subcortical, cerebellar gray matter, brainstem, and ventricle volume with relevance scores from deep-learning-based brain-age predictions across the full lifespan in the LIFE-Adult study (n = 1855).*Top panel*: Region-wise correlation results for the association of subcortical, cerebellar gray matter, brainstem, and ventricle volume with relevance scores; color represents Pearson’s correlation coefficient.*Bottom panel*: Region-wise results for Bonferroni-corrected significant associations of subcortical, cerebellar gray matter, brainstem, and ventricle volume with relevance scores (23 out of 25 regions defined by the FreeSurfer*aseg*atlas).

Out of 25 subcortical regions, including brainstem and cerebellum from the FreeSurfer*aseg*atlas, 19 (all regions except of the ventricles and cerebrospinal fluid; CSF) showed a statistically significant negative relationship between the LRP-based relevance scores and GMV (Bonferroni-corrected, mean*r*= -0.28,*r*-range = [-0.52, -0.14]). The strongest negative correlation was present with the left cerebellar GMV (*r*= -0.52, p < 8.9e-141). Five regions (all of them ventricles or CSF) showed a statistically significant positive relationship (Bonferroni-corrected, mean*r*= 0.44,*r*-range = [0.26, 0.69]). The strongest positive correlation was present in the right lateral ventricle (*r*= 0.69, p < 2.5e-284;Fig. 4).

### 3.3 Cerebral small vessel disease (cSVD) and white matter-related measures

#### 3.3.1 Perivascular spaces (PVS)

In line with previous literature, we found that the number and magnitude of PVS increases with age both in the whole brain (*r*= 0.403, p < 5.3e-74) and areas in and around the basal ganglia (*r*= 0.16, p < 2.9e-12; seeFig. S1in the Supplementary Materials).

**Fig. 5.**
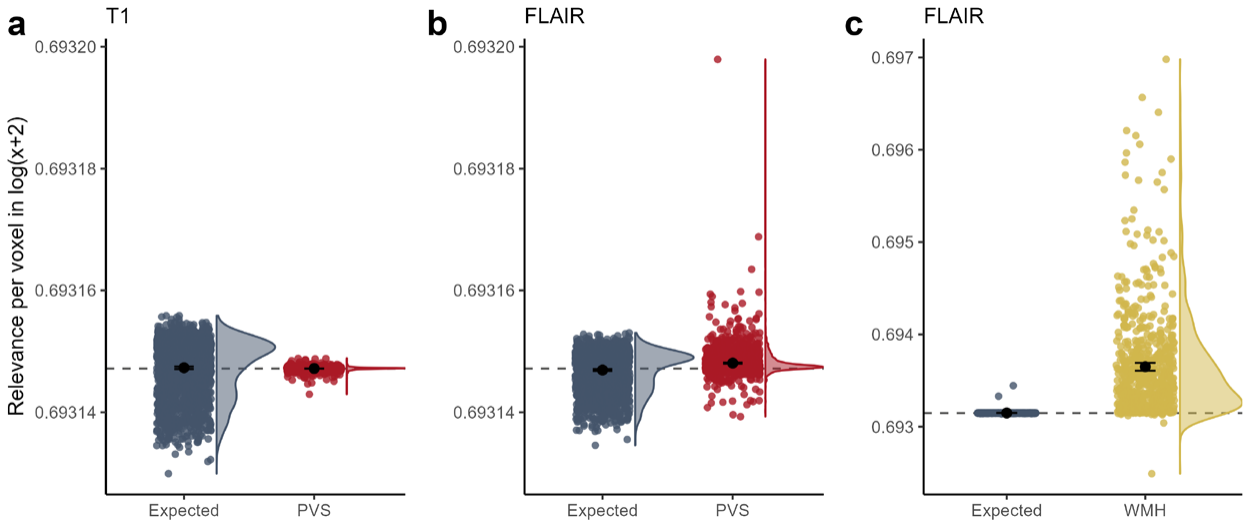
Average relevance in voxels classified as perivascular spaces (PVS). (a) Average relevance in PVS voxels (in red) and expected whole-brain relevance (in blue) in LRP maps from the T1 sub-ensemble (n = 1872). (b) Average relevance in PVS voxels (in red; not dilated) and expected relevance (in blue) in LRP maps from the FLAIR sub-ensemble. (c) Average relevance in white matter hyperintensity (WMH) voxels (in yellow) and expected whole-brain relevance (in blue) in LRP maps from the FLAIR sub-ensemble (reproduced fromHofmann et al., 2022). Relevance scores are shown in log(x+2) scale for better visual representation. Data points were horizontally spread (using*geom_jitter*in*ggplot2)*to further enhance visibility.

There was no significant difference between relevance scores extracted from the T1 sub-ensemble for both PVS maps with no dilation (*d*= 0) and with dilations (*d*in {1, 2}) and the average relevance scores across the brain (one-tailed paired t-tests, no dilation: t*_d_*_=__0_(1871) = -1.17, p*_d_*_=__0_= 0.88,Figure 5a**,**also seeFig. S4ain Supplementary Materials). This was also the case when only considering PVS around BG (one-tailed paired t-test*_d_*_=__1_, t(1871) = -3.85, p = 0.99; t-test*_d_*_=__2_, t(1871) = -5.98, p = 1).

Relevance scores extracted from the FLAIR sub-ensemble were significantly higher in PVS than the relevance score averaged over the whole brain (one-tailed paired t-test, t(1871) = 14.82, p < 2.2e-47, one-tailed;Fig. 5b). However, when using the conservative approach comparing average PVS relevance to the average positive relevance in the whole brain only, no significant difference was found (seeFig. S2bin Supplementary Materials). Also, when only considering PVS around BG, there was no significant difference (paired t-test, t(1871) = 0.284, p = 0.388, one-tailed). InFigure 5c, we show the difference between relevance values in WMH compared to overall positive relevance for comparison, taken fromHofmann et al. (2022).

Upon visual inspection of some individuals with high numbers of PVS, we did not see a systematic correspondence of relevance maps and PVS segmentations, while only some individual PVS were overlapping with higher relevance scores (seeFig. S3in Supplementary Materials).

#### 3.3.2 Fractional anisotropy

**Fig. 6.**
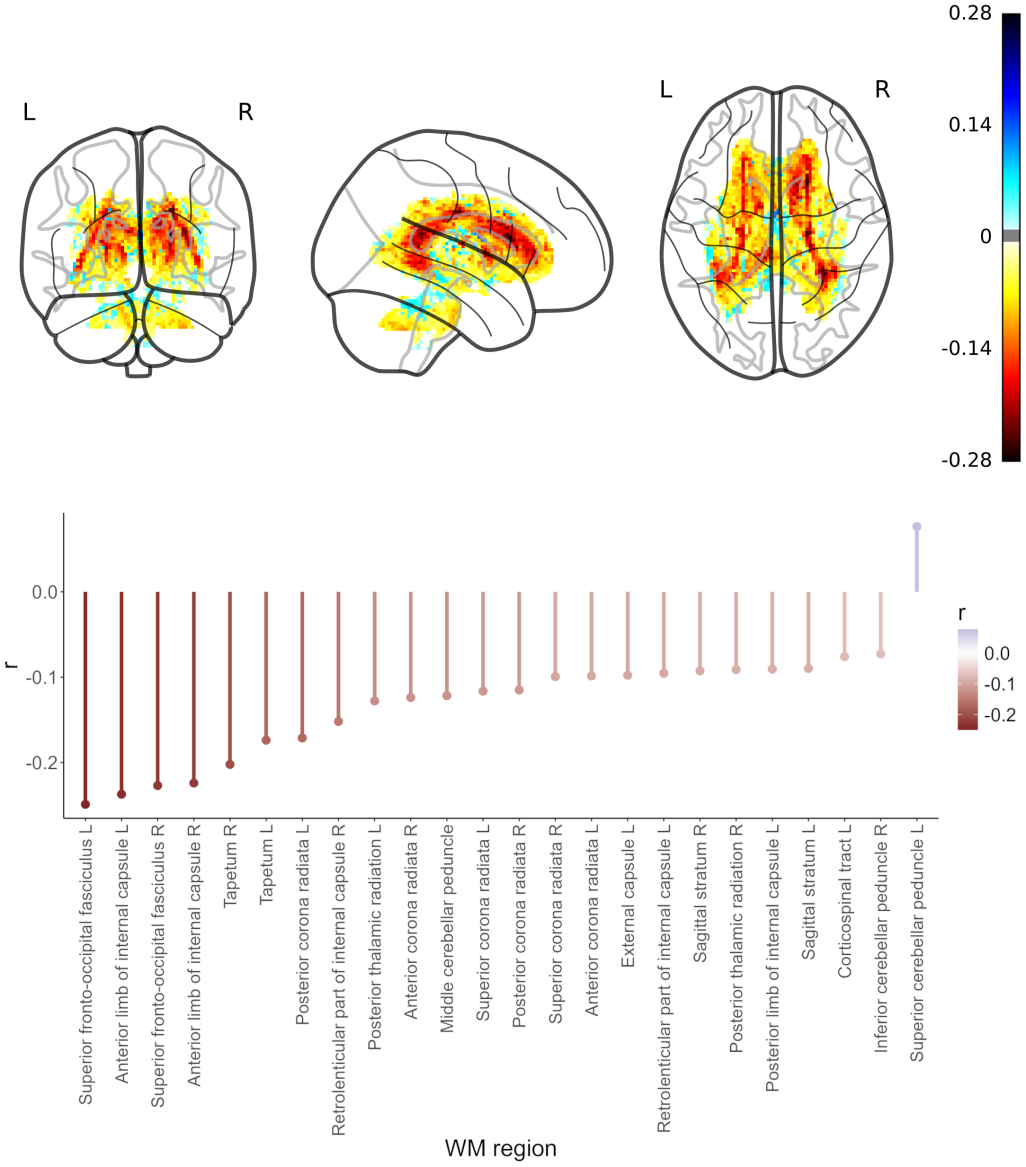
Relationship of fractional anisotropy (FA) with relevance scores from deep-learning-based brain-age predictions across the full lifespan in the LIFE-Adult study (n = 1855).*Top panel*: Voxel-wise correlation results for the association between FA and relevance scores; color represents Pearson’s correlation coefficient.*Bottom panel*: Region-wise results for Bonferroni-corrected significant associations between FA and relevance scores from the FLAIR sub-ensemble (24 out of 48 regions defined by the JHU atlas). Region-wise results for associations between FA and relevance scores from the T1 sub-ensemble are shown inFigure S5in Supplementary Materials.

We performed a region-based and a voxel-wise correlation analysis, investigating the relationship between LRP-based relevance scores and diffusion tensor imaging (DTI)-based fractional anisotropy (FA). For the region-based analysis that related relevance scores from the FLAIR sub-ensemble to FA, we found that in 23 out of 48 regions of the DTI-based WM atlas JHU higher FA values were associated with lower relevance values (Bonferroni-corrected, mean*r*= -0.13,*r*-range = [-0.25, -0.07]). The highest negative correlation was present in the left superior fronto-occipital fasciculus (*r*= -0.25, p < 1.5e-33). For one region, the left superior cerebellar peduncle, lower FA values were associated with lower relevance values (*r*= 0.07, p < 0.01;Fig. 6). The voxel-wise correlation analysis per subject revealed a predominantly negative correlation between FA values and relevance scores (similar to the region-based analysis), with higher FA values associated with lower relevance scores, that is, lower BA (mean*r*= -0.02,*r*-range = [-0.19, 0.09]).

For the region-based analysis that related relevance scores from the T1 sub-ensemble to FA, we found that in 29 out of 48 JHU regions higher FA values were associated with lower relevance values (Bonferroni-corrected, mean*r*= -0.15,*r*-range = [-0.31, -0.05]), similar to the relevance scores from the FLAIR sub-ensemble. The highest negative correlation was present in the right tapetum (*r*= -0.31, p < 2.5e-102). In four regions, lower FA values were associated with lower relevance values (mean*r*= 0.06,*r*-range = [0.05, 0.09];Fig. S5in the Supplementary Materials).

## 4 Discussion

In this work, we showed that DNN-based BA predictions were correlated with widespread differences in common neuroimaging features of aging. In particular, higher XAI-based relevance was associated with lower cortical thickness and volume in frontal-temporal and parietal brain regions. The highest correlation of all features was found between ventricular volume and ventricular relevance. Higher relevance in limbic and basal ganglia regions was also associated with lower GMV. Interestingly, of all non-cerebral features, higher XAI-based relevance in the cerebellum was the most strongly associated with lower cerebellar GMV. Regarding WM differences, we found that higher relevance in frontal-occipital fasciculus and the anterior limb of the internal capsule was strongly negatively related to directedness of WM microstructure in these regions. Contrary to our expectations, PVS were not reliably associated with higher relevance, that is, the DNN models trained on T1 or FLAIR did not consistently consider PVS relevant for predicting BA. Taken together, our method highlighted known imaging features of brain aging, but also highlighted some less considered regions such as the cerebellum.

### 4.1 Relevance of cortical and non-cortical structures for brain age

Our finding of a frontal-temporal parietal pattern of negative associations between relevance and cortical GM features is in line with studies showing linear age associations in superior and inferior frontal gyri, parietal cortex, and superior parts of the temporal lobe (Aycheh et al., 2018;Cox et al., 2021;Fjell et al., 2009;Fjell & Walhovd, 2010). Against our expectation, we found some cortical features in the frontal and temporal lobe to be positively associated with relevance scores. Overall, these associations were weaker than the negative associations and might reflect spurious associations due to mis-segmentations of the pial surface in FreeSurfer. Regarding non-cortical structures, our results of strongest association between ventricular volume and XAI-based relevance confirmed previous findings of the age-related expansion of the ventricular system. We found the largest association for lateral ventricles which showed the second strongest effect of 4.4 % longitudinal annual average change inFjell et al. (2013). We also found that cerebellar GMV among all non-cerebral GM features was the most strongly associated with relevance, which was surprising given that cerebellum GMV showed a lower association with age than, for instance, nucleus accumbens and hippocampus in previous literature (Fjell et al., 2013). FreeSurfer, which was designed to segment the cerebral cortex, might perform suboptimally when determining the GMV of the cerebellum, while the DL model takes an unbiased approach in every region uniquely relying on signal intensity. However, as a recent review suggests, the cerebellum might have a widely underappreciated role in brain aging both structurally and cognitively (Bernard, 2022). In future research, it would be worth exploring the cognitive consequences in participants with particularly high BA relevance scores in cerebellar tissue.

### 4.2 Relevance of perivascular spaces for brain age

We did not find PVS to be consistently considered by the DNNs for brain age prediction. Compared to WMH, PVS, another common imaging marker of cerebral small vessel disease (cSVD), only showed a weak, yet significant increase of FLAIR-based relevance in whole-brain PVS (but not basal-ganglia PVS), and no difference for T1-based relevance maps. Visually we did not see a systematic overlap of relevance values with the PVS segmentation when inspecting individuals with large visible PVS. We, therefore, speculate that the increased FLAIR-based relevance might have been confounded by two factors: First, some of the deep WM PVS considered were close to the cortical surface, which showed consistently higher relevance values both based on T1 and FLAIR sub-ensembles (seeHofmann et al., 2022). Second, WMH are known to form around PVS (Wardlaw et al., 2020), which could result in higher FLAIR relevance attributed to PVS. From a mathematical point of view, PVS in T1w-images should be surrounded by higher relevance values rather than overlapping with them, given that the BA model considers them as relevant. This is because only non-zero image intensity values can contribute to the model estimates of BA and PVS or other fluid-containing spaces usually have zero-intensity values in T1w images. We, therefore, dilated the PVS masks to detect surrounding relevance scores in T1-based relevance maps. However, in contrast to FLAIR-based maps for T1-based relevance maps, we did not find any difference between PVS and all other voxels, even though PVS are commonly detected on T1w images (Duering et al., 2023). This was the case no matter whether original or dilated PVS maps were used, indicating that the shape or spatial features of this structure were not taken into account by the DNNs. For areas in and around the basal ganglia, we found that the association between age and PVS volume was smaller as compared to whole-brain PVS (Fig. S1in the Supplementary Materials); therefore, we can also expect a smaller relationship between BA relevance scores and PVS in these regions.

As the two cSVD imaging markers WMH and PVS tend to co-occur (Wardlaw et al., 2020), the lack of consideration of the DNNs for PVS might indicate that WMH and PVS are not sufficiently distinct (i.e., their appearance might be topologically collinear) to provide additional information to the model. Previous research also demonstrated that the relationship between PVS and age is modulated by other features such as intracranial volume and hypertension (Huang et al., 2021). Lastly, although we did find a robust correlation between the total number of PVS voxels and age (Fig. S1in the Supplementary Materials), the relationship with PVS may be too subtle (Barisano et al., 2021;Lynch et al., 2023) to be captured by our BA model, when compared to more salient aging processes in the brain.

### 4.3 Relevance of white matter structures for brain age

We found that higher FA values in periventricular frontal and occipital WM were the most strongly negatively associated with attributed XAI-based relevance. This finding reflects the strong association of aging with WMH, and their highest prevalence in periventricular WM (De Leeuw et al., 2001). We showed previously that the average relevance in WMH was significantly larger than the average positive relevance outside WMH (Hofmann et al., 2022). Here, we used relevance based on DNN-models trained on FLAIR, which reflect signal intensity differences related to WMH. Diffusion-related imaging metrics are known to be severely altered in WMH, displaying reduced FA and increased mean diffusivity which reflects increased water content, and in severe cases damaged fiber tracts in these regions (Wardlaw et al., 2015). With our DNN models trained on FLAIR, which is primarily susceptible to tissue water content, we could not quantify the contribution of other subthreshold WM differences which might appear from a model trained on diffusion-weighted imaging. Interestingly, we also found a relationship between FA values and relevance scores obtained from the T1 sub-ensemble in approximately 69% of the WM regions. The regions with the highest negative correlation with FA showed a strong overlap between FLAIR and T1-based relevance maps. While it is known that T1 values are altered in severe WMHs (Maniega et al., 2015), our results suggest that the T1 sub-ensemble might be capable of detecting more subtle changes in T1 intensities to predict brain aging. This is consistent with findings showing that T1w signal intensity is influenced by microstructural properties like myelin content, which are closely related to FA through their shared sensitivity to tissue integrity and anisotropy (Chang et al., 2017;Koenig, 1991).

### 4.4 Limitations

So far, we could not provide an indication to which extent further unknown imaging features contribute to the BA estimation for the 3D-DNNs. We did not directly compare FA values with relevance based on the same imaging modality, and the association with FLAIR-based relevance might have underestimated the association with age as FLAIR is not susceptible to diffusion-based differences except the impact of tissue water. In future work, an additional sub-ensemble could be attached to the BA model architecture where DTI is utilized for model training and, subsequently, XAI-based relevance maps can be computed directly from the DTI model branch.

Also, relevance maps of LRP should be carefully evaluated. First, they could be selectively interpreted to confirm*a priori*assumptions leading to a confirmation bias (Ghassemi et al., 2021). We, therefore, chose to report results across the whole brain, avoiding selective ROIs. Second, not all relevance values within a map must be meaningful with respect to the predicted (target) variable; hence, one must assume a certain level of noise in these maps. This can be illustrated by untrained neural networks that are often sensitive toward strong image contrasts, resulting in spurious saliency maps (Adebayo et al., 2020). We, therefore, chose group-level statistics to average out noise effects over participants. Particularly for within-subject analyses, one could consider recently promoted techniques for DNN-based modeling that mitigate potential confounding effects resulting from preprocessing steps such as skull stripping and image registration (Tinauer et al., 2022). Third, while we controlled for the size of different parcels in our region-based analyses using the sum over the average of relevance per region, we ignored more detailed distributions of relevance scores within these regions. Fourth, LRP (and similar XAI approaches) highlight only voxel-wise contributions to the model estimates, while underlying relationships between voxels can only be inferred with domain knowledge. There are new variants of LRP that allow to extract higher-order structure (Eberle et al., 2022;Schnake et al., 2022) or underlying relevant concepts (Achtibat et al., 2023; here, e.g., this could be interacting structural changes of two brain regions); hence, it would be a promising path of research to employ Concept Relevance Propagation (seeTinauer et al., 2024).

## 5 Conclusion

Taken together, we show that highly accurate DNN-based multi-modal BA predictions are partially driven by morphometric changes in the cortex, subcortex, and WM. While most age-related cortical thinning, subcortical GM atrophy, ventricular expansion, and WMH appearance have been investigated earlier, we showed that these relationships can be discovered by end-to-end models, that is, these models are not explicitly trained on these features but trained on relatively unprocessed MR images to estimate age. At the same time, also less considered regions such as the cerebellum are highlighted by this data-driven approach.

End-to-end DNNs generally have higher predictive accuracy compared to linear models or other classic machine-learning approaches that are trained on pre-selected features (e.g., see the brain-age competition PAC 2019). Here, we have provided evidence that DNNs consider these biologically relevant brain features. However, their high prediction performance was only partially explained by these features. In future work, our explainable AI pipeline could help to identify unknown brain changes, which could be further enhanced by high-resolution imaging.

## Supplementary Material

Supplementary Material

## Data Availability

Due to potential identifiability of individuals from demographic and medical information, we cannot share the processed data used in this study. Raw data of the LIFE-Adult cohort can be requested via the LIFE study center (https://ldp.life.uni-leipzig.de/). All code is inhttps://github.com/SHEscher/RelevanceRelated.
