# Supplementary material for: The utility of explainable AI for MRI analysis: Relating model predictions to neuroimaging features of the aging brain
Source: Imaging Neurosci (Camb). 2025 Feb 27;3:imag_a_00497. doi: 10.1162/imag_a_00497 (PMC12319959; doi:10.1162/imag_a_00497)
Supplement: Supplementary Material [file imag_a_00497-supp.pdf]

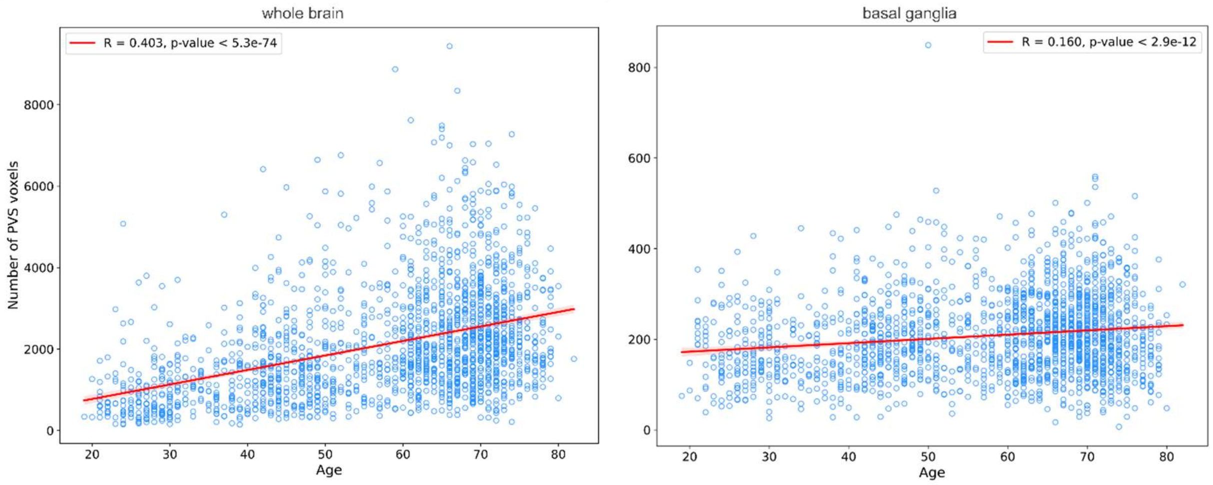

Fig.S1: Relationship between age and perivascular spaces (PVS) in the whole brain (*left*) and in the area in and around the basal ganglia (*right panel*). PVS segmentation was done using SHIVA-PVS toolbox (Boutinaud et al., 2021; https://github.com/pboutinaud/SHIVA_PVS; accessed in March 2024). The basal ganglia was defined by the ATAG atlas (https://www.nitrc.org/projects/atag), and dilated by 5 voxels to include deep white matter areas surrounding the basal ganglia. *Red line*: Pearson correlation *R* between age and number of PVS voxels across participants.

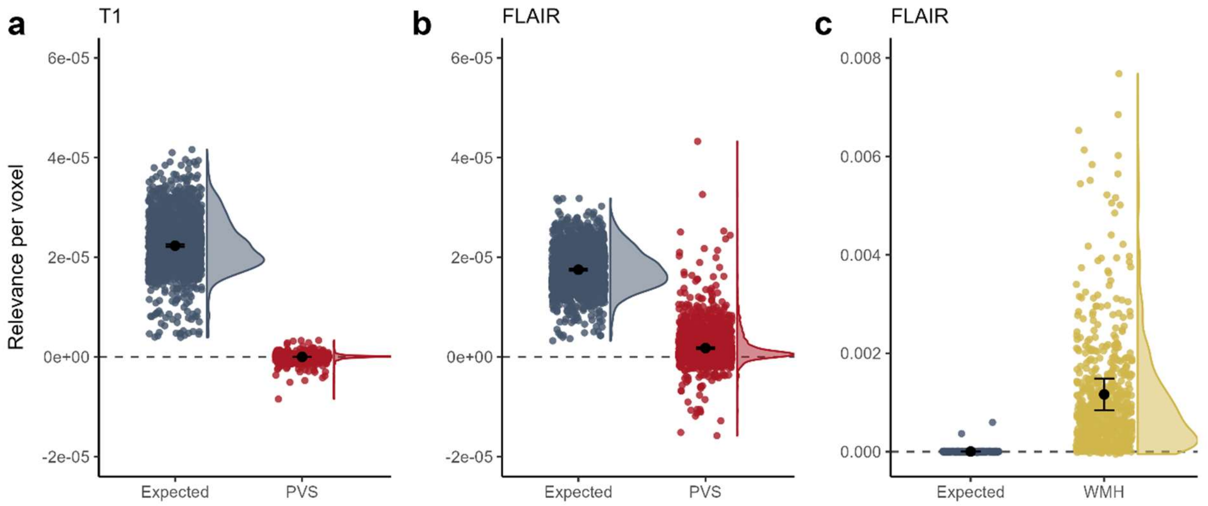

Fig.S2: Average relevance in voxels of perivascular spaces (PVS) compared to average relevance of voxels with positive relevance. (**a**) Average relevance in PVS voxels (in red) and expected relevance (in blue) for LRP-heatmap of the T1 sub-ensemble (n = 1855). (**b**) Average relevance in PVS voxels (in red) and expected relevance (in blue) for LRP-heatmap of the FLAIR sub-ensemble. (**c**) Average relevance in WMH voxels (in yellow) and expected relevance (in blue) for LRP-heatmap of the FLAIR sub-ensemble (reproduced from Hofmann et al. (2022). Here, expected relevance is defined as the average relevance score of all positive relevance voxels in the whole brain for the corresponding participant. This is a more conservative comparison level, as compared to all relevance voxels as a baseline. Data points were plotted using *geom_jitter* in *ggplot2* to enhance visibility.

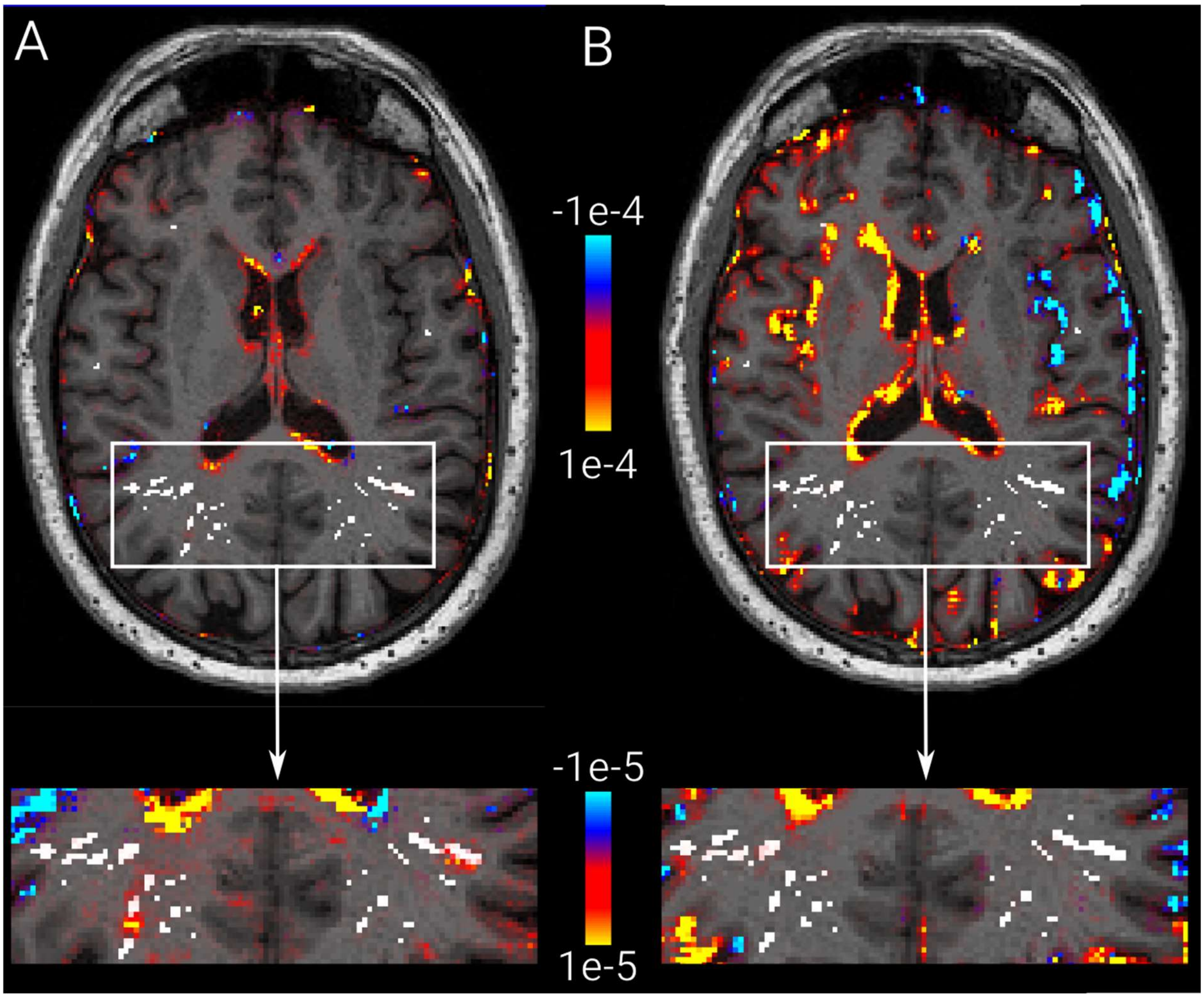

Fig.S3: Perivascular space (PVS) segmentation and brain age relevance in a 65-year old male. A: PVS segmentation (white) and relevance values from LRP based on the T1 sub-ensemble overlaid on the corresponding T1w image. **B**: PVS segmentation (white), relevance values from LRP based on FLAIR sub-ensemble. (upper panels: red/blue: positive/negative relevance thresholded at 10^{-4}, lower panels: relevance thresholded at 10^{-5})

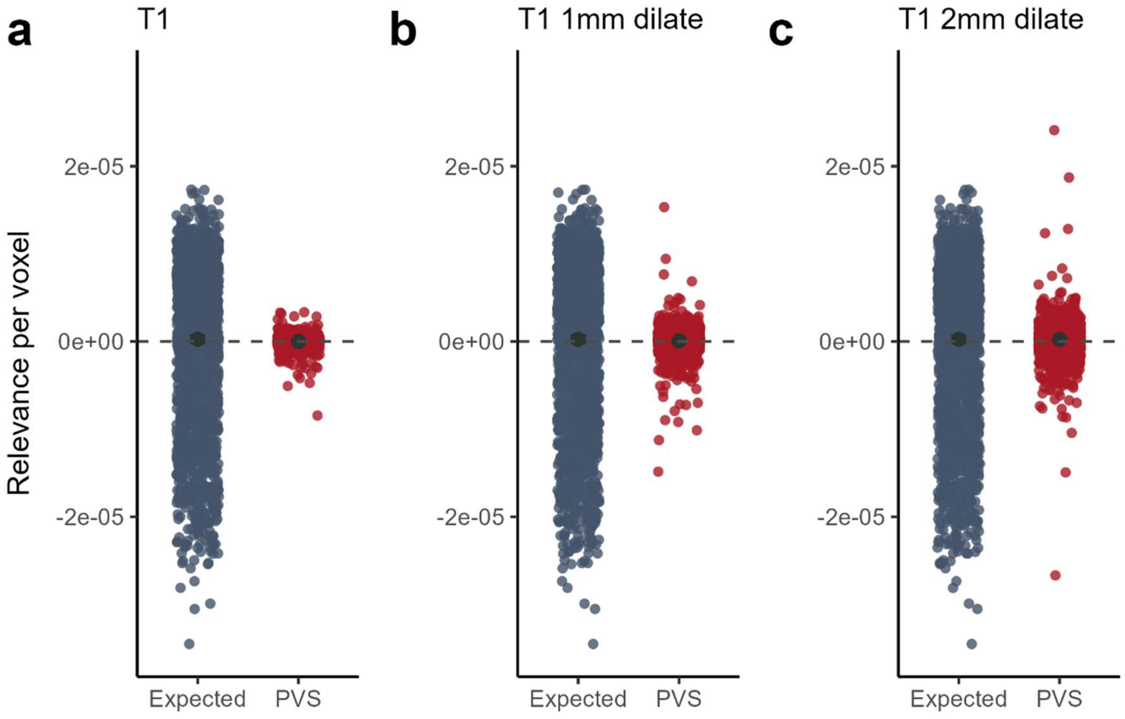

Fig.S4: Effect of dilation on T1 sub-ensemble based relevance values of PVS. (**a**) Average relevance in PVS voxels (in red) and expected relevance (in blue) for LRP-heatmap of the T1 sub-ensemble (n = 1855). (**b**) Average relevance in PVS voxels (in red) and expected relevance (in blue) for LRP-heatmap of the T1 sub-ensemble with 1 mm dilation, t-test: td=1(1871) = -0.93, pd=1 = .82.(**c**) Average relevance in PVS voxels (in red) and expected relevance (in blue) for LRP-heatmap of the T1 sub-ensemble with 2 mm dilation, t-test: td=1(1871) = -0.058, pd=1 = .52. For none of the three, the relevance in PVS is statistically significantly greater than the expected relevance. Data points were plotted adding small random variation to the location of each datapoint to enhance visibility (using *geom_jitter* in *ggplot2*).

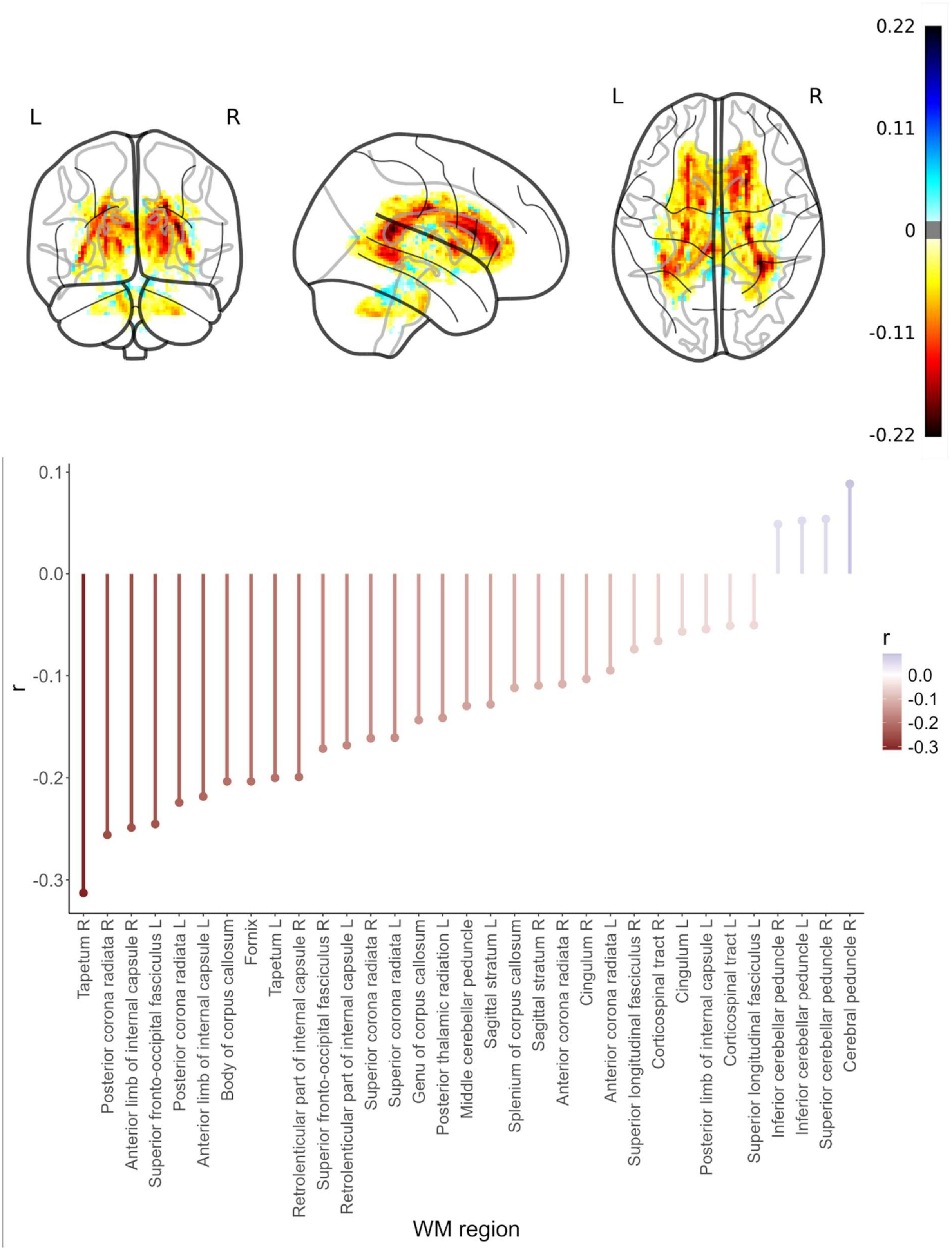

Fig.S5: Relationship of regional fractional anisotropy (FA) with relevance scores obtained from T1 sub-ensemble LRP heatmap across the full lifespan in the LIFE-Adult study (n = 1855). Only Bonferroni-corrected statistically significant associations are shown (33 out of 48 regions defined by the JHU atlas).
